# Immunomodulatory Protein Hydrolysates and Their Application

**DOI:** 10.3390/nu10070904

**Published:** 2018-07-14

**Authors:** Mensiena B. G. Kiewiet, Marijke M. Faas, Paul de Vos

**Affiliations:** Immunoendocrinology, Division of Medical Biology, Department of Pathology and Medical Biology, University Medical Center Groningen, University of Groningen, Hanzeplein 1, 9700 RB Groningen, The Netherlands; m.m.faas@umcg.nl (M.M.F.); p.de.vos@umcg.nl (P.d.V.)

**Keywords:** protein hydrolysate, bioactive peptide, immunomodulation, Toll-like receptor, functional foods

## Abstract

Immunomodulatory protein hydrolysate consumption may delay or prevent western immune-related diseases. In order to purposively develop protein hydrolysates with an optimal and reproducible immunomodulatory effect, knowledge is needed on which components in protein hydrolysates are responsible for the immune effects. Important advances have been made on this aspect. Also, knowledge on mechanisms underlying the immune modulating effects is indispensable. In this review, we discuss the most promising application possibilities for immunomodulatory protein hydrolysates. In order to do so, an overview is provided on reported in vivo immune effects of protein hydrolysates in both local intestinal and systemic organs, and the current insights in the underlying mechanisms of these effects. Furthermore, we discuss current knowledge and physicochemical approaches to identify the immune active protein sequence(s). We conclude that multiple hydrolysate compositions show specific immune effects. This knowledge can improve the efficacy of existing hydrolysate-containing products such as sports nutrition, clinical nutrition, and infant formula. We also provide arguments for why immunomodulatory protein hydrolysates could be applied to manage the immune response in the increasing number of individuals with a higher risk of immune dysfunction due to, for example, increasing age or stress.

## 1. Introduction

**Figure 1:**
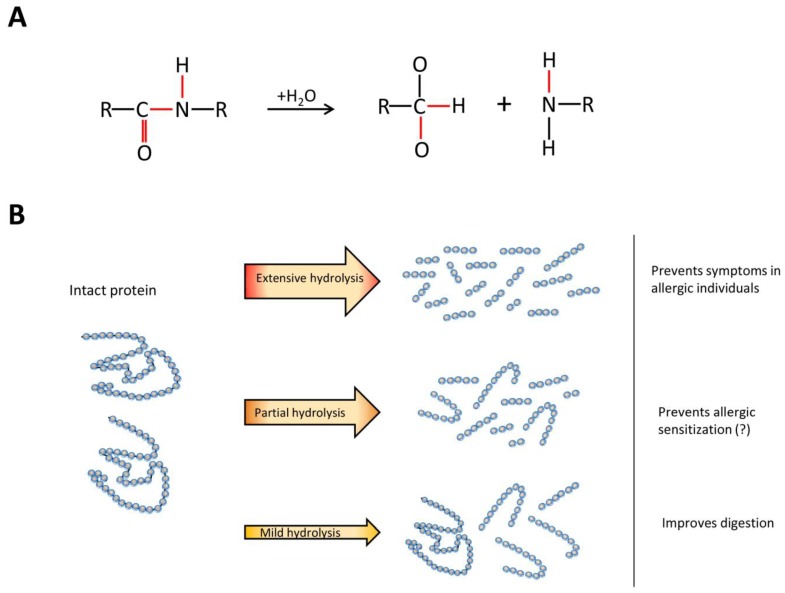
The process of protein hydrolysis and its products. (**A**) chemical reaction of protein hydrolysis; (**B**) different hydrolysates serve different purposes.

Protein hydrolysates are commonly used as an alternative protein source in commercial products. They consist of a mixture of different proteins and peptides which is formed by the hydrolysis of intact proteins. During this process, peptide bonds of intact proteins are broken (Figure 1A), which results in the formation of a range of peptides of different sizes. Depending on their properties, protein hydrolysates are applied in different products. Mildly hydrolyzed proteins are, for example, used in clinical and sport nutrition to support digestibility, while extensively hydrolyzed proteins are used in infant formulas as a hypo-allergenic alternative for intact cow’s milk proteins (Figure 1B).

Furthermore, protein hydrolysates are recognized as a potent source of bioactive peptides. Different peptides with, for example, anti-thrombotic, anti-hypertensive, anti-microbial, anti-cancer, anti-oxidative, and many immunomodulatory effects have been identified [1]. Consuming protein hydrolysates containing these peptides might be helpful in the management of many western diseases [2,3]. Since many of these diseases are immune-related, immunomodulatory products have gained special attention from both academical and industrial researchers for the management and amelioration of, for example, inflammatory bowel diseases, allergies, and diabetes [4,5].

Purposively deploying the immunomodulating effects of protein hydrolysates in existing or new dietary products is an attractive opportunity to manage immune-related diseases. In order to achieve this, the identification of specific peptides and a better understanding of their working mechanisms is required. This review aims to provide an overview of studies on reported in vivo immune effects of hydrolysates in both local intestinal and systemic organs. The current insights in underlying mechanisms are also discussed. As the design of effective protein hydrolysates may benefit from the identification of specific bioactive peptides, we review current knowledge and physicochemical approaches to identify the protein sequence(s). Based on the discussed topics, we provide our view on the possible application of immunomodulatory protein hydrolysates or peptides in specific target groups.

## 2. Immune Effects of Hydrolysates

In 1984, immune effects were detected for the first time in a fraction of a casein hydrolysate [6]. The studied protein fraction was found to both increase the production of hemolytic antibodies in mice splenocytes, and to enhance the phagocytic capacity of murine macrophages against sheep red blood cells in vitro. In vivo, the protein fraction protected mice against a lethal infection with *Klebsiella pneumoniae*. Since then, measuring lymphocyte proliferation and macrophage phagocytosis capacity have been the main in vitro assays used to study the immune effects of protein hydrolysates.

An increase in lymphocyte proliferation was observed after stimulation with other protein hydrolysates which were derived from soy, wheat, whey, casein, and mollusk [7,8,9,10,11,12,13]. Not all protein hydrolysates were found to stimulate proliferation. Hydrolysates derived from egg white were shown to decrease instead of enhance lymphocyte proliferation [14]. Different hydrolysates from casein were found to possess either proliferation increasing [10] or inhibiting effects [15], showing that individual protein hydrolysates from the same protein source also have remarkably different immunomodulating properties. Phagocytosis modulation was source dependent as well. Most protein hydrolysates were found to increase macrophage phagocytosis capacity in vitro, for example, soy, egg, wheat, and casein hydrolysates had such an effect [9,12,16,17]. However, a rice protein hydrolysate was found to inhibit the phagocytic activity of RAW264.7 macrophages [18].

**Figure 2:**
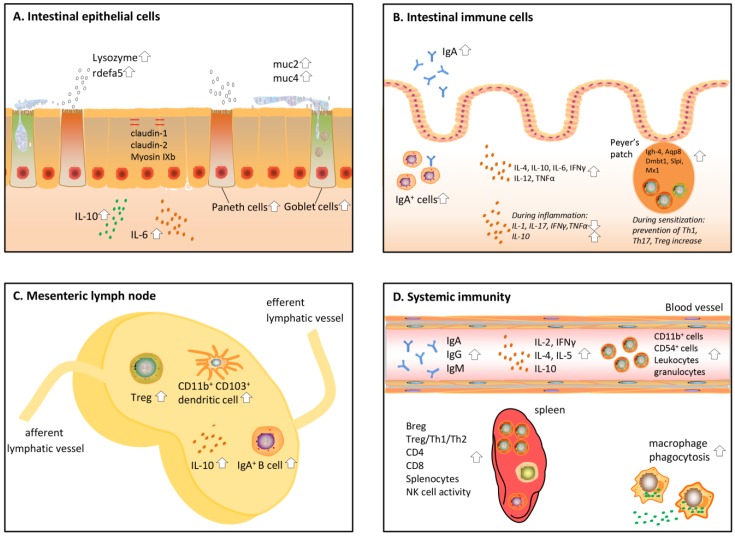
Overview of immune effects induced by protein hydrolysates on the (**A**) intestinal epithelial cells; (**B**) intestinal immune cells; (**C**) mesenteric lymph nodes; (**D**) systemic immune system.

These early in vitro experiments investigating the immune effects of protein hydrolysates have led to many more in vitro and in vivo studies on the effects of protein hydrolysates on immunity. In the sections below, these in vivo studies are reviewed (an overview of the studies is given in Table 1). In this part, a distinction was made between local intestinal immune effects and systemic effects. All effects discussed below are also visualized in Figure 2. To start with, protein hydrolysates have an effect on the epithelial cells aligning the gut and by that induce crosstalk between the epithelial cells and immune cells [5].

## 3. Effects of Protein Hydrolysates on the Gut Barrier

Epithelial cells form a first layer of defense against harmful pathogens and molecules in the intestinal lumen. These cells are covered by a layer of mucus. Together, this forms a physical barrier between the luminal content and the human body [46]. Protein hydrolysates and bioactive peptides in the lumen of the intestine were found to affect barrier function in multiple ways (Figure 2A). First, protein hydrolysates are able to strengthen the epithelial barrier. Second, they enhance the production of mucus and so-called anti-microbial proteins that delete pathogens.

Barrier enhancing effects by protein hydrolysates were shown by Visser et al., [19,20], who fed diabetes prone rats casein hydrolysates for up to 150 days and measured the barrier function by determining the lactulose-mannitol ratio in urine. They showed that casein hydrolysate intake decreased the epithelial permeability compared to a diet with sole amino acids. In the ileum of diabetic rats on the casein hydrolysates, a normalization of the tight junction mRNA expression, including myosin IXb, claudin-1, and claudin-2, was observed, together with an upregulation of the regulatory cytokine Interleukin (IL)-10. Multiple other dairy derived peptides have been found to increase the amount of goblet and Paneth cells in the intestine [21,22,23], which are specialized epithelial cells regulating the production of mucus and anti-bacterial peptides. The consumption of yoghurt peptides did not only increase the amount of these cell types, but also increased the expression of Muc2 and 4, as well as the anti-bacterial factors lysozyme and rdefa5 [21].

Another function of epithelial cells is sampling the lumen and skewing the differentiation of immune cells in the lamina propria accordingly, mainly by basolateral cytokine secretion [47]. In a healthy situation, egg yolk peptide digest consumption in mice was able to increase IL-6 production in epithelial cells ex vivo [24]. IL-6 has been described to affect both innate and adaptive immune responses [48,49], meaning that protein hydrolysates might influence the immune system via epithelial cells in this way. However, due to the pleiotropic nature of this cytokine [50], its impact is different in different cell types and conditions, and the overall effect of increased IL-6 is difficult to predict.

## 4. Effects of Protein Hydrolysates on the Intestinal Immune System

One of the most studied immune effects in the intestine after hydrolysate administration in vivo is the level of IgA (Figure 2B). This typical intestinal immunoglobulin can easily be measured in the faeces and is instrumental in the clearance of toxins, pathogens, and other harmful molecules [51]. Hydrolysate intake can cause an increase in IgA, as shown for a common carp egg hydrolysate [25]. Corresponding to this, studies investigating intestinal immunity after pacific whiting, shark hydrolysate, and yellow field pea hydrolysate consumption in mice detected an increased amount of IgA^+^ B cells [26,27,28]. Leblanc et al. studied IgA secretion in an infection mouse model fed *Lactobacillus helveticus*-fermented milk, and found an increase of IgA both in the intestinal fluid and blood, together with an increased amount of IgA^+^ cells in the lamina propria [29]. Increased IgA levels seem to be a general effect of hydrolysate intake and seem to be independent of the hydrolysate protein source.

Besides enhancing IgA production, protein hydrolysates have more effects on the immune system which might lead to a more matured and developed immune response. Levels of multiple cytokines, including IL-4, IL-10, IL-6, IFNγ, IL-12, and TNFα, have been found to be elevated in healthy mice after consuming egg yolk, fish, and yellow pea hydrolysate [24,26,27]. For the shark hydrolysate, these increased cytokine levels were associated with a better response of the mice against *E. Coli* infection [28]. The enhancement of IL-4, IL-10, IFNγ, and IL-12 suggests that T cells in the intestine are activated by the hydrolysate. To understand what the administration of protein hydrolysates could mean in situations such as pathogenic infections or inflammatory bowel disease, it would be helpful to know which types of T cells are stimulated. To this end, Leblanc et al., compared the amount of IL-4 (Th2) and IFNγ (T helper (Th)1) in the intestine of a mouse model fed *Lactobacillus helveticus*-fermented milk, and found a predominant Th2 response after feeding the milk peptides [29].

Anti-inflammatory properties are another often observed feature of protein hydrolysates. This is a characteristic mainly attributed to the hydrolysates of bovine milk. To study these effects, experimental models with colitis and ileitis have been used. In all cases, oral pretreatment of the animals with casein hydrolysate or a casein glycomacropeptide reduced damage to the intestine, leading to a decrease in weight loss [30,31,32,33]. They also observed a decrease in the pro-inflammatory cytokines IL-1β, IL-17, TNFα, and IFNγ, while in one study, an increase of the regulatory cytokine IL-10 was observed [31]. Similar outcomes were observed when egg white peptides were administered to pigs with dextran sodium sulphate (DSS) induced colitis [34].

The effects of protein hydrolysates in the Peyer’s patches (PP), which are part of the gut-associated lymphoid tissue, have been studied to a lesser extent but are essential as this tissue is the central immune sampling and signaling site in the gut. Egusa et al. studied the effects of a specific soybean protein digest in the PP [35]. After five weeks of feeding mice a soybean hydrolysate enriched diet, the genome wide gene expression of PP derived cells was examined. They found that several genes related to innate immunity and host defense were upregulated. They observed the upregulation of *Igh-4* and *Aqp8*, which enhance phagocytosis, and of Dmbt1, *Slpi*, and *Mx1,* which are anti-bacterial and anti-viral components [52,53,54]. When looking at adaptive responses in the PP, we found in our own study that sensitization with a partially hydrolyzed whey protein prevented the increase of Th1, Th17, and regulatory T cells (Treg) in the PP after a challenge with intact whey [36].

## 5. Effects of Protein Hydrolysates on the Mesenteric Lymph Nodes (MLN)

Tolerance induction and other antigen presenting dependent processes occur predominantly in the MLN [55]. Antigen loaded dendritic cells from the PP and lamina propria in the intestine migrate to the MLN and subsequently activate T and B cells. Depending on the nature of the antigen, either an immune response is evoked or tolerance is induced via the formation of Treg. A number of studies investigating the effects of bovine milk hydrolysates or peptides in murine sensitization models assessed which cell types were present in the MLN of the mice after hydrolysate consumption (Figure 2C). When mice were fed milk peptides, either alone or in combination with indigestible oligosaccharides, before the start of the sensitization against intact whey, the number of Treg in the MLN increased [37,38]. One study showed that the number of dendritic cells responsible for the transport of antigens from the lamina propria to the MLNs, i.e., the CD11b^+^ and CD103^+^ dendritic cells, was increased [37]. Casein derived glycomacropeptide consumption increased ex vivo tolerogenic IL-10 production in MLN cells of a DSS induced colitis mouse model [32], which also suggests an increase in Treg here. However, in a rat model for ileitis, the glycomacropeptide did not influence MLN cytokine levels [33]. This might be explained by differences in species, inflammation models, or glycomacropeptide dosing.

Not only T cell responses are induced in the MLN, but also B cells can be activated, after which they expand in specialized follicles. It was found that sensitization with a partially hydrolyzed whey protein increased the number of IgA^+^ B cells [36]. However, since the role of the MLN in intestinal IgA production is still under debate, more research is needed to evaluate the contribution of this observed phenomenon to intestinal and systemic IgA levels [56].

## 6. Effects of Protein Hydrolysates on Systemic Immunity

Protein hydrolysates do not only affect the immune response locally in the intestine, but also have effects on the systemic immunity, mostly measured in splenic and peritoneal cells, and in the blood (Figure 2D). Small peptides are probably able to pass through the gut barrier and have been detected in the blood of volunteers who consumed dairy and soy products, [57,58] allowing a direct impact on immune cells in the systemic circulation. Administration of many protein hydrolysates made from casein and other milk products, oyster, salmon, and fish [17,27,39,40,41] increased the ex vivo phagocytotic capacity of macrophages isolated from the peritoneal cavity of mice. Oyster, salmon, and common carp egg were furthermore found to increase NK cell activity in the spleen [25,39,41].

Similar to results after direct in vitro stimulation of splenocytes, oral administration of protein hydrolysate derived from oyster, tuna cooking drip, salmon, and multiple common carp egg hydrolysates increased ex vivo splenocyte proliferation [25,39,41,42]. Some of these studies looked into the cell types present in the spleens of these animals in more detail. Alcalase common carp egg hydrolysate was found to increase CD4^+^ and CD8^+^ cells in the spleen [25], while salmon hydrolysate only increased CD4^+^ [41]. These cells were expected to be both Th1 and Th2 cells, since both Th1 (IL-2, IFNγ) and Th2 (IL-4, IL-5) cytokines were detected in the blood of these animals. These effects might differ between protein hydrolysates, since a tuna cooking drip hydrolysate was found to increase IL-2 and IL-10 [42], and sensitization with a partial whey hydrolysate increased Treg in the spleen [36].

Together with an increase in Treg, an increase in regulatory B cells (Breg) was observed in the spleen after whey hydrolysate administration [36]. Breg are increasingly recognized to be important in regulatory immune responses and induce the differentiation of T cells into Treg [59]. Another study also found an increase in IL-10 producing Breg when inducing oral tolerance using intact casein in casein-allergic mice [60]. Here, casein might be digested in the intestine of the mice, after which the newly formed bioactive peptide(s) derived from casein increased Breg. An adoptive transfer of these Breg could even prevent the onset of allergy in recipients [60], demonstrating the importance of this cell type in tolerance induction.

Other evidence that hydrolysate consumption affects B cell responses is the observation of increased antibody levels observed in the blood. A soy protein hydrolysate was found to induce IgG and IgA in the blood of rats [7], while the common carp egg hydrolysate increased IgA in mice [25]. Therefore, it is likely that protein hydrolysates not only affect B cell differentiation, but also induce class-switching and antibody production.

Only a few studies investigated the effects of protein hydrolysates on immune parameters in the blood of humans. The effect of a single dose of soybean hydrolysate was studied in a small group of volunteers, and it was found to change leukocyte numbers and increase granulocytes. More specifically, it significantly increased CD11b^+^ (macrophages and/or dendritic cells) and CD56^+^ cells (NK cells) in blood [43]. When nine subjects were fed a wheat hydrolysate for six days, an increase in NK cell activity was observed [44]. A larger group of undernourished Indian children was given a fish hydrolysate for 120 days. After this period, the CD4/CD8 ratio and antibody levels were measured, but no significant differences were detected [45]. These studies show that protein hydrolysates have immunomodulatory effects in humans, which are similar to some of the previously mentioned in vitro and in vivo effects, although the protein hydrolysates used are different. However, in these studies, only a few immunological parameters were measured, and/or only included a small group of volunteers, which makes it difficult to draw firm conclusions. Well-designed, extensive human studies are lacking at the moment, but are needed to better understand the effects of protein hydrolysates in humans.

## 7. Understanding Hydrolysate Compositions

A mandatory assignment for the coming years for researchers in the field is to identify the exact peptide sequence(s) responsible for the reported effects described above. This will make the design of formulations possible for specific health effects. Currently, protein hydrolysates are mainly characterized by determining their degree of hydrolysis (DH). The DH is a measure for how extensively a protein has been hydrolyzed, and therefore gives an indication of the size of the peptides present in the hydrolysate. The DH is calculated by determining the amount of cleaved peptide bonds [61]. Different methods have been developed to obtain the DH, including the pH-stat, trinitrobenzenesulfonic acid (TNBS), o-phthaldialdehyde (OPA), trichloroacetic acid soluble nitrogen (SN-TCA), and formol titration methods [62].

However, the DH does not give any indication of the presence or absence of specific bioactive peptides. Protein hydrolysates with a similar DH can still have a different peptide composition. Therefore, protein hydrolysates are often further characterized by using techniques to separate the peptides in the hydrolysate based on their physicochemical properties, mainly size, hydrophobicity, or a combination thereof. Techniques which are often used to characterize peptide composition are, for example sodium, dodecyl sulfate polyacrylamide gel electrophoresis (SDS-PAGE), size exclusion chromatography, and High Performance Liquid Chromatography (HPLC) [63]. All these techniques are used to obtain a molecular weight distribution, which gives an overall indication of the hydrolysate composition based on size. One can focus on a specific fraction of the protein hydrolysates by choosing the most appropriate technique and optimizing the settings used.

HPLC, especially Reverse Phase-HPLC, has, for a long time, been found to be useful in separating peptides from protein hydrolysates based on their size and hydrophobicity [64]. When this method is coupled to a mass spectrometer (MS), it is also possible to determine the amino acid sequence of the detected peptides [65]. In this way, a very detailed characterization of the hydrolysate can be obtained, which can be used to identify structure-effector relationships between, for example, immune effects and specific peptides in a hydrolysate. However, protein hydrolysates consist of thousands of different peptides, which will all be detected by HPLC-MS when a complete hydrolysate is analyzed. Therefore, studies aiming for the identification of bioactive peptides often compare the bioactivity of size-based fractions of the hydrolysate first [66,67], after which the bioactive fraction alone can be further analyzed using HPLC-MS. Ultimately, the peptides present in the bioactive fraction should be tested individually for bioactivity in order to obtain the peptide responsible for the observed effect [65].

Using this and similar methods has led to the discovery of a range of bioactive peptides from protein mixtures, as reviewed by Sanchez et al., and Lafarga et al., [1,68]. However, most of the identified bioactive peptides possess anti-hypertensive, anti-microbial, or anti-oxidative effects, but peptides with immunomodulatory properties have not widely been identified yet. Comparing the characteristics of the immunomodulatory peptides that have been identified generated new knowledge about which peptide properties are associated with immune effects. It is known that immunomodulatory peptides are mostly two to 20 amino acids long and hydrophobic [61]. Chalamaiah et al., concluded by listing known peptides with immunomodulatory effects that glycine (Gly), valine (Val), leucine (Leu), proline (Pro), phenylalanine (Phe), negatively charged amino acid glutamic acid (Glu), and aromatic amino acid tyrosine (Tyr) were most frequently present in peptides with immune effects [69].

Recently, we also found that larger fractions in whey and soy protein hydrolysates can have immunomodulatory effects. These fractions have a size of over 1000 kDa and were composed of aggregates which are formed during the hydrolysis process [66]. In this process, the proteins are heated, which is a known cause of protein denaturation and aggregation [70]. By performing PAGE under different conditions, it was found that these aggregates were formed due to electrostatic forces and disulfide bridges between single proteins and induce responses in human dendritic cells. The fact that these aggregates were found in both a whey and soy hydrolysate suggests that aggregate formation is not protein source specific and might be present in a wide range of different protein hydrolysates.

## 8. Underlying Mechanisms of Immunomodulatory Effects

**Figure 3:**
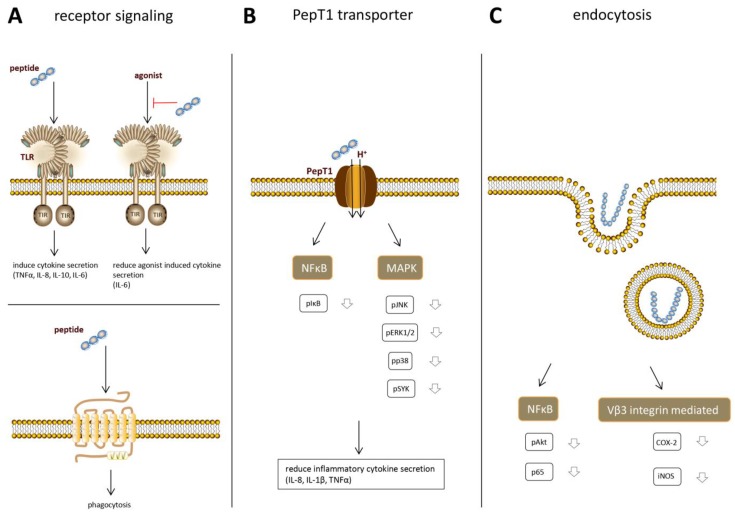
Overview of mechanisms described in the literature via which peptides can exert immunomodulatory effects in the cell. Peptides can (**A**) directly stimulate receptors; (**B**) be taken up in the cell via a peptide transporter and interfere with inflammatory signaling pathways; or (**C**) be taken up into the cell via endocytosis and inhibit inflammatory signaling pathways.

In order to understand the immune effects that can be induced by protein hydrolysates and to ultimately apply protein hydrolysates in specific conditions, it is of crucial importance to understand exactly how the observed immune effects come about. Up to now, only a few studies have focused on elucidation of the underlying mechanisms of immunomodulatory effects by protein hydrolysates and are reviewed below. Recent research suggests that hydrolysate peptides bind to specific immune-receptors and that multiple receptors might be involved (Figure 3A).

## 9. Receptor Binding

One of the most studied receptor types in immune signaling is Toll-like receptors (TLRs). This is a family of pathogen recognition receptors. They are not only expressed by most immune cells [71,72], but also by epithelial cells [73]. Multiple protein hydrolysates were found to affect TLRs, but the effects were very hydrolysate dependent. By studying a range of cow’s milk hydrolysates in a TLR reporter cell platform, we previously found that especially mildly hydrolyzed whey hydrolysates were able to activate multiple TLRs, including TLR2, 3, 4, 5, 7, 8, and 9 [74]. This activation does lead to the production of TNFα, IL-10, and IL-8 in human peripheral blood mononuclear cells (PBMCs). The protein source of the hydrolysates was crucial for its final effects, as it was observed that casein hydrolysates only inhibited TLR activation. Which TLRs were inhibited also differed per hydrolysate, but TLR5 and 9 were the most profoundly inhibited. Other studies focused mainly on TLR2 and 4. Tobita et al., described that a casein phosphopeptide was able to induce proliferation and IL-6 production in CD19^+^ cells from mice after stimulation in vitro. This effect was gone after the administration of an anti-TLR4 antibody, suggesting that the effects were induced via TLR4 [75]. A primary culture of murine intestinal epithelial cells was also thought to secrete IL-6 via the stimulation of both TLR2 and TLR4 when the mice were fed with yellow pea hydrolysate [26] and shark protein hydrolysate [28]. A pressurized whey hydrolysate was able to suppress lipopolysaccharide (LPS) induced IL-8 production in respiratory epithelial cell lines, likely via binding to TLR4 [76].

There is evidence that other transmembrane receptors are also involved in immune effects by protein hydrolysates. Tsuruki et al., described that a peptide derived from the soybean β-conglycinin A’ subunit, which stimulated phagocytosis in human neutrophils, showed a low affinity for the *N*-formyl-methionyl-leucyl-phenylalanine (fMLP) receptor [77]. The phagocytosis stimulating effect disappeared when this receptor was blocked. The authors discuss that its low affinity for the fMLP receptor allows it to stimulate the immune response in a safe way, without causing inflammation.

Peptides derived from many different food protein sources are known to bind opioid receptors [78]. Although endogenous opioid peptides have a main function as neurotransmitters, they are also known to modulate innate and acquired immune responses [79]. Opioid receptor signaling can, for example, skew T cell differentiation, increase antibody production in B cells, and affect phagocytosis in macrophages [80,81,82]. These effects have also been described in immune cells after hydrolysate administration. Therefore, it cannot be excluded that protein hydrolysates also modulate the immune system via opioid receptors.

## 10. PepT1 Dependent Intracellular Effects

Multiple bioactive tri- and tetrapeptides derived from soy and whey have been found to induce anti-inflammatory effects after being taken up into the cell [83,84,85]. This cellular uptake, and therefore the anti-inflammatory effects of the peptides, depends on the peptide transporter PepT1 (Figure 3B). PepT1 is an H^+^ coupled oligopeptide transporter, mediating the uptake of a broad range of di-and tripeptides in intestinal epithelial cells in order to transport the peptides into the bloodstream. Normally, it is expressed in the small intestine, but during inflammation, it is also upregulated in the colon [86]. Treatment with the soy peptides KVP and VPY and the whey peptide IPAV all showed a decrease in the production of the pro-inflammatory cytokines IL-6, IL-8, and TNFα in Caco2 cells. This effect disappeared when PepT1-activity was inhibited, indicating that transport via PepT1 is necessary for the anti-inflammatory effects. Once taken up in the cytosol, the peptides were shown to inhibit the main inflammatory signaling pathways in order to decrease the pro-inflammatory cytokine secretion. A decrease in phosphorylated nuclear factor kappa-light-chain-enhancer of activated B cells (NFκB), mitogen-activated protein kinase (MAPK), extracellular signal-regulated kinase (ERK)1/2, c-Jun N-terminal kinase (JNK)1/2, p38, and spleen tyrosine kinase (SYK) was observed after pretreatment with the bioactive peptides.

As described above, gut epithelial cells can influence the immune response in the intestine [47]. Therefore, an anti-inflammatory status of the epithelial cells is expected to also regulate the underlying immune cells [87]. Furthermore, the soy and whey proteins can be transported over the epithelial barrier and interact directly with immune cells. The soy peptide VPY was also found to induce anti-inflammatory effects in THP-1 human monocytes [84], while the soy peptide KVP showed anti-inflammatory effects in T cells [83]. Interestingly, both monocytes and T cells express the PepT1 transporter [88], suggesting that the bioactive peptides might induce anti-inflammatory effects in innate and adaptive immune cells via a similar mechanism as described for epithelial cells.

The relevance of these effects in vivo was shown by treating mice with DSS and TNBS induced colitis with the bioactive soy peptides [83,84]. In these models, KVP and VPY were shown to reduce colitis symptoms, reduce body weight loss, and decrease pro-inflammatory cytokines in the intestine.

## 11. Endocytosis

Once immunomodulatory peptides are taken up in epithelial or immune cells, they can exert their effects by interfering with signaling pathways. However, not all peptides and protein can be internalized via the PepT1 transporter, since this transporter is specific for di- and tripeptides. Larger food derived peptides, which are too large for the PepT1 transporter, can also be taken up into the cell by fluid phase endocytosis (Figure 3C), which is a non-specific form of vesicle mediated internalization. In this type of endocytosis, hydrophobic interactions between the peptide and the cell membrane are involved in the internalization of the peptide [89]. Differences in physicochemical properties of peptides, including size, hydrophobicity, and charge determine the kinetics of uptake of individual peptides.

The involvement of this type of peptide uptake in immunomodulation by peptides was confirmed by Regazzo et al., [90]. They showed that a relatively large, hydrophobic casein peptide which showed multiple stimulating effects in immune cells, could be taken up in a layer of Caco2 cells via endocytosis. They did not see a difference in casein peptide uptake when they used an inhibitor for the PepT1 transporter or cytochalasin D to open the tight junctions (and increase the paracellular route), but found a significant inhibitory effect on peptide uptake after treatment with wortmannin, which inhibits endocytosis [90]. When the peptide is translocated over the epithelial cells via this mechanism, it may affect immune cell functioning in the lamina propria or in the blood.

A study investigating the well-characterized soy peptide lunasin showed that endocytosis is also used by immune cells to take up immunomodulatory peptides [91]. Lunasin is a 43-amino acid peptide which was shown to interact with the αVβ3 integrin, which led to inhibiting αVβ3 integrin-mediated pro-inflammatory markers and to downregulation of the Akt-mediated NF-κB pathway. By using different inhibitors, it was found that lunasin was mainly taken up by endocytic mechanisms that involve integrin signaling, clathrin-coated structures, and macropinosomes [91]. Interestingly, this lunasin uptake was increased under inflammatory conditions.

## 12. Possibilities for Hydrolysate Application

Since immune effects were found to be hydrolysate specific, many immune-related conditions could potentially benefit from hydrolysate administration by selecting specific protein hydrolysates. However, up to now, research has mainly focused on the discovery of immune effects in vitro and animal studies, while follow up studies in humans are rare. To develop an immunomodulatory product containing a hydrolysate, these studies are indispensable in order to investigate the safety, bioavailability, and inducible immune effects in the human body. Protein hydrolysates with the most promising effects should be chosen for further research. Based on the knowledge on immune effects and the underlying mechanisms involved, of which an overview was given above, protein hydrolysates can be selected for application in specific products.

## 13. Existing Products

**Figure 4:**
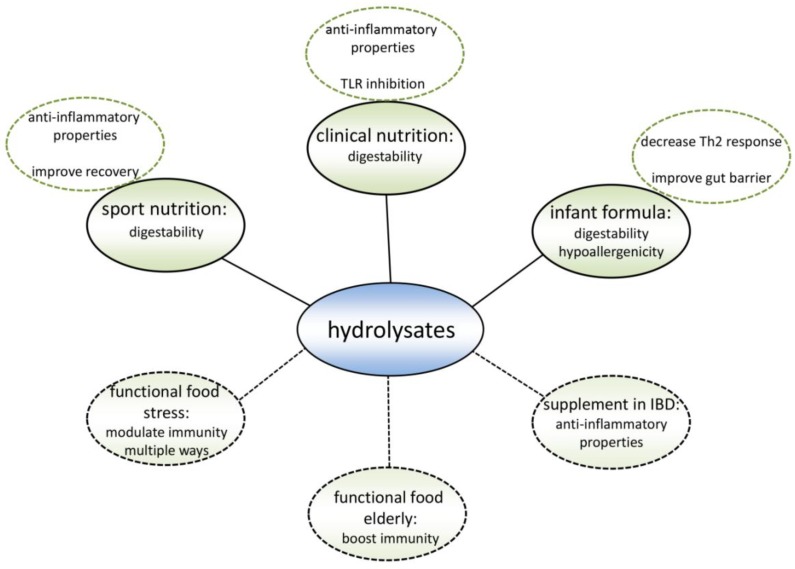
Summary of the application possibilities of protein hydrolysates. These hydrolysates are currently being used in sport nutrition, clinical nutrition, and infant formula, mainly because of their good digestibility and hypoallergenicity. Recent research indicates that specific protein hydrolysates could optimize the current products in multiple ways. Also, there is evidence that new protein hydrolysate products could be beneficial for specific target groups.

**Figure 5:**
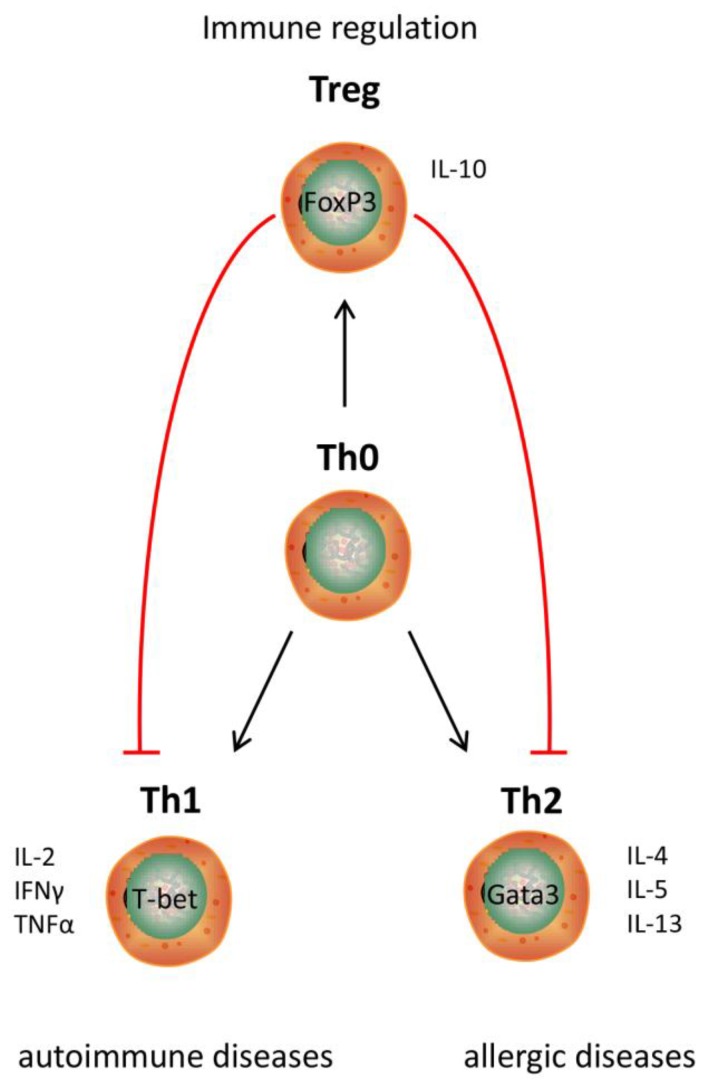
Overview of T helper cell subsets and their interactions and their relation to specific diseases.

Existing hydrolysate containing products can benefit from applying a different protein hydrolysate with the same nutritional value as the currently used protein hydrolysates, but with an additional immune modulating effect (Figure 4). Hypo-allergenic infant formulas are the main market for protein hydrolysates nowadays. By hydrolyzing proteins, epitopes which are recognized by the immune system of allergic infants are destroyed. Therefore, infant formulas containing extensively hydrolyzed proteins can be tolerated by allergic infants [92]. However, allergic reactions may also be reduced by modulating the immune system [5]. When the allergic reaction develops, the intestinal immune system induces an inappropriate immune response against a harmless food molecule [93], which is characterized by an increased Th2 response [94] (Figure 5). Since multiple cow’s milk hydrolysates have been described to induce Treg cell differentiation in the MLN and spleen [36,38] which reduces the Th2 response [95], these protein hydrolysates might help to reduce allergic symptoms.

T cell differentiation can be regulated via TLRs [96,97]. Therefore, protein hydrolysates might affect the T cell response via TLRs, as they have been found to modulate TLR signaling [74]. This knowledge could help in selecting allergy reducing protein hydrolysates, since TLR signaling of protein hydrolysates can be measured in reporter cell platforms [74]. Furthermore, protein hydrolysates have also been found to reduce the permeability of the intestinal epithelial barrier [19]. This might provide an alternative mechanism for reducing the allergic reaction, since it reduces the uptake of antigens and prevents the interaction of lamina propria immune cells with antigens.

Another product in which protein hydrolysates are already used is clinical nutrition. In general, a high protein intake was found to decrease mortality in hospitalized patients [98]. Protein hydrolysates are used instead of intact proteins because of their ease of digestion. Anti-inflammatory protein hydrolysates might have an additional benefit in patients consuming clinical nutrition because of intestinal inflammation, because many studies have found beneficial effects of anti-inflammatory protein hydrolysates in multiple colitis mouse models [30,31,32,33]. Therefore, anti-inflammatory protein hydrolysates may be expected to reduce symptoms of, for example, inflammatory bowel disease and irritable bowel syndrome in patients.

Intestinal inflammation is also a common side-effect of chemotherapy [99]. Therefore, individuals undergoing chemotherapy could be another target group for anti-inflammatory protein hydrolysates. Interestingly, some chemotherapeutic agents induce inflammation via specific TLRs [100,101]. During chemotherapy, intestinal epithelial cells are damaged, which leads to the release of Damage associated molecular patterns (DAMPs). DAMPs are able to activate TLR activation, which initiates an inflammatory response [102,103]. Therefore, inhibiting TLR signaling protects against chemotherapy induced inflammation. It was indeed found that the intake of food molecules could reduce the inflammation caused by the chemotherapeutic agent doxorubicin, by the inhibition of TLR2 [104]. Since protein hydrolysates can also inhibit TLRs [74], they might elicit similar effects.

Protein hydrolysates are also applied in sport nutrition. Proteins are known to be essential in the recovery after exercise [105], and peptides are more rapidly taken up compared to intact protein [106]. Reducing inflammatory responses was found to reduce muscle pain and aid in the recovery after exercise. This can be done by taking anti-inflammatory food products, for example, tart cherry juice, which decreased pain and increased muscle recovery after running long distances when ingested prior to the exercise [107,108]. Protein hydrolysates with an anti-inflammatory effect could therefore simultaneously provide a protein source needed for muscle anabolism, and aid in the recovery due to anti-inflammatory effects. Some studies indeed suggest that the administration of whey protein hydrolysate or wheat gluten hydrolysate prior or after exercise decreased muscle damage, improved muscle repair, and improved the performance [109,110,111]. Furthermore, excessive exercise may result in impaired immunity [112]. Immune modulating protein hydrolysates could also be beneficial for athletes in this respect.

## 14. Target Groups for New Products

Protein hydrolysates could also be used to develop completely new products with immunomodulatory effects (Figure 5). Many studies showing a new immunomodulating effect of a hydrolysate suggest that the hydrolysate could be used as a nutraceutical or functional food. Both terms are used for food products with an additional health effect besides their nutritional value. As outlined in the introduction, we and others feel that immunomodulating products will be particularly useful in modern Western society, since the occurrence of immune-related diseases is currently increasing [113].

As mentioned before, protein hydrolysates have a good chance of ameliorating symptoms in local intestinal diseases like inflammatory bowel disease and irritable bowel syndrome. In addition, patients suffering from systemic immune diseases are likely to benefit from hydrolysate consumption. Type 1 diabetes is an example of an autoimmune disease with a rapidly increasing prevalence [114]. Animals studies suggest that casein hydrolysate can prevent autoimmune diabetes by modulating multiple immune responses in the intestine [19,20]. Management of autoimmunity to delay or prevent disease may therefore be another promising field of application for specific protein hydrolysates.

In our view, some specific target groups might also specifically benefit from specific immunomodulatory protein hydrolysates in order to delay or prevent disease. The number of elderly in the population is increasing. With increasing age, the immune system deteriorates and becomes more proinflammatory. This leads, for example, to more infections, and therefore a higher mortality rate [115]. The innate immune response is affected in multiple ways during immunoscenesence. An altered cytokine production by monocytes and macrophages has been found, together with a decreased phagocytotic capacity and a reduced TLR expression [116,117]. Since it was found that specific protein hydrolysates are able to modulate these immune aspects [27,74], protein hydrolysates might be beneficial in keeping the elderly healthy for a longer period of time. This improves quality of life and reduces health care costs.

Stress is another common cause of immune dysfunction [118]. Currently, an increasing number of people experience significant levels of stress, leading to more immune-related diseases [119,120]. Both acute and chronic stress has been found to induce immune dysfunction, resulting in inflammatory, autoimmune, and allergic diseases [121,122]. Effects associated with the development of immune diseases due to stress are increased pro-inflammatory cytokines, more Th2-related cytokines, and changes in leukocyte number and distribution [123,124,125,126]. As described above, specific protein hydrolysates are able to counteract these effects. Therefore, functional food containing protein hydrolysates may contribute to healthy immunity in people experiencing significant stress levels.

## 15. Conclusions

A wide range of protein hydrolysates have immunomodulatory capacities. However, before protein hydrolysates can serve as functional foods, physicochemical approaches to identify the protein sequence(s) are needed to be able to design effective protein hydrolysates. Also, specific target groups have to be identified. In this way, specific protein hydrolysates can be designed to ameliorate, delay, or prevent the onset of a wide variety of Western immune-related conditions.

## Figures and Tables

**Table 1:** Overview of hydrolysates and their immune effects.

Hydrolysate/Peptide	Enzyme/Treatment	Immune Effects	Species	Reference
Casein hydrolysate diet (200 g/kg casein, TD99482, Harlan-Teklad Custom Research, Madison, WI, USA	Not applicable (NA)	Reduction of autoimmune diabetes by 50%, decreased lactulose/mannitol ratio, decreased serum zonulin levels, increased ileal TEER, altered ileal mRNA expression of Myo9b, claudin-1, and claudin-2.	Diabetes prone BB rat	[19]
Casein hydrolysate (20% of diet Pancase S^TM^ (Sensient Flavours, Strassbourg, France) or Nutramigen^TM^ (Mead Johnson Nutrition, Zeeland, MI, USA))	NA	Reduction of autoimmune diabetes, decreased lactulose/mannitol ratio, increased ileal IL-10 levels, beneficial gut microbiota changes (increased *Lactobacilli* and reduced *Bacteroides* spp. levels)	Diabetes prone BB rat	[20]
β-CN(94-123) from commercial yoghurt	NA	Enhanced numbers of goblet and Paneth cells in the small intestine, increased expression of Muc2, Muc4, lysozyme, and rdefa5.	rat	[21]
Yoghurt or Milk Fermented by *Lactobacillus casei* DN-114 001	*Lactobacillus casei* DN-114 001	Increased cell proliferation and villous area in the proximal intestine, hypertrophy and hyperplasia of Paneth and goblet cells.	mouse	[22]
Milk fermentation products of *L. Helveticus* R389	*L. Helveticus* R389	Enhanced expression of calcineurin in the small intestine, upregulated IL-2 and TNF production, increased number of mucosal mast cells and goblet cells	mouse	[23]
Egg yolk digests	Pepsin	Increase of the IL-6 secretion by small intestinal epithelial cells, increase in IgA^+^ cells, orchestrating the Th1/Th2 response.	mouse	[24]
Common carp egg hydrolysate	Pepsin, alcalase	Increase of secretory immunoglobulin A in the gut. Pepsin hydrolysate increased the splenic NK cell cytotoxicity, macrophage phagocytosis and level of serum immunoglobulin A (IgA). S-IgA in the gut was significantly enhanced by pepsin and alcalase hydrolysates. Trypsin hydrolysate increased the percentages of CD4^+^ and CD8^+^ cells in the spleen.	mouse	[25]
Yellow field pea hydrolysate	Thermolysin	Increased number of IgA^+^ cells in the small intestine lamina propria, accompanied by an increase in the number of IL-4^+^, IL-10^+^, and IFNγ ^+^ cells.	mouse	[26]
Fermented pacific whiting protein	Yeast	Enhanced phagocytic activity of peritoneal macrophages, increased number of IgA^+^ cells, and increased IL-4, IL-6, IL-10, IFNγ, and TNFα levels in the small intestine lamina propria	mouse	[27]
Shark protein hydrolysate Peptibal^TM^ (innoVactiv Inc)	Trypsin and chymotrypsin	Increase of small intestinal immunoglobulin A-producing cells and intestinal IL-6, TNFα, TGFβ, and IL-10	mouse	[28]
Peptide fraction from *Lactobacillus helveticus*-Fermented Milk	Lactobacillus Helveticus	Increased intestinal and serum IgA levels, increase in the number of IgA-secreting B lymphocytes in the intestinal lamina propria, stimulation of Th2 response (IL-4 vs. IFNγ)	mouse	[29]
κ-casein–derived glycomacropeptide	NA	Decreased body weight loss, decreased anorexia, colonic damage, a reduction in colonic alkaline phosphatase activity, IL-1, trefoil factor 3, and iNOS mRNA levels.	Rat (TNBS induced colitis)	[30]
β-Casein hydrolysate	Cell envelope-associated proteinase of Lactobacillus delbrueckii ssp. lactis CRL 581	Decreased mortality rates, faster recovery of initial body weight loss, less microbial translocation to the liver, decreased β-glucuronidase and myeloperoxidase activities in the gut, decreased colonic macroscopic and microscopic damage, increased IL-10 and decreased IFNγ.	Mouse (TNBS induced colitis)	[31]
κ-casein–derived glycomacropeptide	NA	Rag1^-^/^-^:increased body-weight gain, decreased colonic damage score and myeloperoxidase (MPO) activity, reduced percentage of CD4^+^ interferon IFNγ^+^ cells and increased IL-6 in MLN. Increased colonic expression of TNFα and IFNγ and increased IL-10 in MLN, by MLN. DSS: decreased MPO activity, increased IL-10 production in MLN.	Mouse (DSS induced colitis and Rag1^-^/^-^)	[32]
bovine glycomacropeptide	NA	Decrease of inflammatory injury, as assessed by lower extension of necrosis and damage score, myeloperoxidase, alkaline phosphatase, inducible nitric oxide synthase, IL- 1β, TNFα, and IL-17.	Rat (TNBS induced colitis)	[33]
Egg white hydrolysate	Aminopeptidase	Attenuated DSS-induced clinical symptoms, including weight loss, mucosal and submucosal inflammation, crypt distortion, and colon muscle thickening, and decreased intestinal permeability and increased mucin gene expression, reduced intestinal expression of pro-inflammatory cytokines TNFα, IL-6, IL-1β, IFNγ, IL-8, and IL-17.	Pig (DSS induced colitis)	[34]
Soybean protein hydrolysate	Rhizopus oryzae neutral protease preparation	Increased number of IL-12^+^CD11b^+^ in spleens, increased cytotoxic activity of spleen cells, increased *Igh-4*, *Aqp8*, *Dmbt1*, *Slpi*, and *Mx1* in Peyer’s patch cells.	Mouse	[35]
Partially hydrolyzed whey protein	NA	Increased Breg and Treg in the spleen, increased IgA^+^ B-cells in the MLN, increased Th1, activated Treg and activated Th17 cells in the Peyer’s patches	Mouse	[36]
LLDAQSAPLRVYVEELKP (from whey)	NA	Reduced acute allergic skin response, decreased whey-specific antibody levels, increased the percentages of CD11b^+^CD103^+^ dendritic cells and CD25^+^Foxp3^+^ T cells in the MLN.	Mouse	[37]
Partial whey hydrolysate	NA	Reduced acute allergic skin response and mast cell degranulation after whey challenge, increased Foxp3^+^ regulatory T-cell numbers in the MLN.	Mouse	[38]
oyster peptide-based enteral nutrition formula	Bromelain, pepsin, trypsin	Enhanced spleen lymphocyte proliferation and of NK cell activity	Mouse	[39]
Casein hydrolysate	Trypsin	Phagocytosing capacity of phagocytic cells was increased	Mouse	[17]
Milk protein hydrolysate		Improved the level of hemolysin in serum, and enhanced phagocytosis of macrophages. In ovalbumin-sensitized mice, the milk protein hydrolysates reduced IgE levels, reduced IL-4 in serum, reduced the release of histamine and bicarbonate in peritoneal mast cells, and enhanced TGFβ levels.	ICR mouse	[40]
Chum salmon oligopeptide preparation	Complex protease	Enhanced lymphocyte proliferation capacity increased number of plaque-forming cells, increased NK cell activity, increased percentage of CD4^+^ T helper (Th) cells in spleen and secretion of Th1 (IL-2, IFNγ) and Th2 (IL-5, IL-6) type cell cytokines.	ICR mouse	[41]
Tuna cooking drip hydrolysate	Enzyme A and B	Increased weight of the spleen and thymus and enhanced the proliferation of splenocytes. Increased production of IL-10 and IL-2. Increased serum IgG1 and IgG2a levels.	Mouse	[42]
Soy protein hydrolysate	Pepsin	Increased serum IgA and IgG levels	Rat	[7]
Soy protein hydrolysate	Theroase, bioprase, Sumizyme FP	Total lymphocyte and granulocyte numbers were altered, and the numbers of CD11b^+^ cells and CD56^+^ cells increased.	Human	[43]
Wheat gluten hydrolysate	NA	NK cell activity increased significantly	Human	[44]
Fish protein hydrolysate (Amizate)	NA	No effects observed	Human	[45]
